# Continuity of Care in Adults Aging with Cerebral Palsy and Spina Bifida: The Importance of Community Healthcare and Socioeconomic Context

**DOI:** 10.3390/disabilities3020019

**Published:** 2023-06-12

**Authors:** Anam M. Khan, Paul Lin, Neil Kamdar, Elham Mahmoudi, Philippa Clarke

**Affiliations:** 1 Institute for Social Research, University of Michigan, Ann Arbor, MI 48106, USA; 2 Institute for Healthcare Policy and Innovation, University of Michigan, Ann Arbor, MI 48109, USA; 3 Center for Disability Health and Wellness, University of Michigan, Ann Arbor, MI 48108, USA; 4 Department of Surgery, Michigan Medicine, University of Michigan, Ann Arbor, MI 48109, USA; 5 Department of Obstetrics and Gynecology, Michigan Medicine, University of Michigan, Ann Arbor, MI 48109, USA

**Keywords:** aging, disability, congenital, continuity of care, healthcare, environment, community

## Abstract

Continuity of care is considered a key metric of quality healthcare. Yet, continuity of care in adults aging with congenital disability and the factors that contribute to care continuity are largely unknown. Using data from a national private administrative health claims database in the United States (2007–2018). we examined continuity of care in 8596 adults (mean age 48.6 years) with cerebral palsy or spina bifida. Logistic regression models analyzed how proximity to health care facilities, availability of care providers, and community socioeconomic context were associated with more continuous care. We found that adults aging with cerebral palsy or spina bifida saw a variety of different physician specialty types and generally had discontinuous care. Individuals who lived in areas with more hospitals and residential care facilities received more continuous care than those with limited access to these resources. Residence in more affluent areas was associated with receiving more fragmented care. Findings suggest that over and above individual factors, community healthcare resources and socioeconomic context serve as important factors to consider in understanding continuity of care patterns in adults aging with cerebral palsy or spina bifida.

## 1. Introduction

“Aging with Disability” is a term that refers to people living with the long-term effects of disabilities acquired early in life who are now surviving into mid- and later life [1]. Increased life expectancy among those with disabilities acquired at birth (e.g., cerebral palsy, spina bifida) is attributed to advancements in medicine, technology, and public health [2,3]. From a life course perspective, individuals aging with disability experience the dynamic processes of aging superimposed on their disability, putting them at risk of worse health outcomes compared to those who develop a disability in later life [1].

Evidence suggests that adults aging with disability exhibit signs of accelerated or “premature” aging and are likely to enter mid- to late-life in worse health than the general population [1,4]. Despite sparse data at the population level in the United States, clinical and survey research indicate that chronic health conditions in people aging with disability typically occur about 20–25 years sooner than those without disability [5]. Compared to those without disability, individuals aging with disability experience higher rates of chronic disease, worse self-rated health, and premature mortality [6-9]. Additionally, there are risks of developing secondary health conditions, such as increased spasticity, osteoporosis and osteoarthritis that originate directly or indirectly from the primary disability [10-12]. Thus, individuals aging with a physical disability have complex healthcare needs, including appropriate care for their primary disability, routine preventive services (e.g., screenings), and care for chronic and secondary conditions, that require ongoing medical monitoring and coordinated care management over time [13,14].

Continuity of care (COC) across settings and over time is considered a key metric of many care delivery models [15-17], especially for older adults with multiple chronic conditions that require comprehensive medical management [18]. For adults aging with disability, complex care needs must be met by professionals with a range of skills and specialties [13], and continuous care reflects the degree to which these services are delivered in coordinated and uninterrupted succession over time [19]. There is ample evidence that people with disabilities receive poor standard healthcare, including disparities in screening and preventive services, cancer diagnosis and treatment, reproductive and pregnancy care, communication with health care professionals, and satisfaction with care [14,20-25]. People with disabilities also encounter a multitude of environmental barriers to accessing care, including lack of transportation and distance to treatment centers, which create challenges for continuous care delivery [21,26-28]. Thus, proximity to healthcare facilities, availability of primary care and specialist providers, and access to transportation are critical for supporting continuous care for people aging with disability. Yet, COC in adults aging with disability and the community factors that contribute to care continuity in this population are largely unknown.

Person-level factors associated with high COC in the general population have been well characterized, including older age, female sex, white race and fewer comorbid health conditions [29,30]. There is also emerging evidence on the role of the community environment in care continuity amongst individuals with psychiatric disabilities, where a greater density of mental health centers and practicing psychologists were associated with more continuous care [31]. However, for individuals aging with a physical disability, there may be additional community features that are important for accessing care, including public transit and broadband internet access (for telehealth), and access to tertiary care specialists [22,32-34].

In order to address the limitations in the current state of knowledge, the current work leverages data from a large nationwide medical claims database to examine COC in a cohort of adults aging with cerebral palsy and spina bifida in the United States. Cerebral palsy (CP) is the most common pediatric onset disability with increased survival in recent decades [35], and spina bifida (SB) is a congenital birth defect that often results in severe life-long disability and morbidity [9]. Individuals with CP and SB have significant and progressive motor impairment, excessive sedentary behavior, inadequate muscle and bone development, and are at risk of secondary chronic disease as they age [36,37]. In this study we provide one of the first estimates of COC in adults aging with CP or SB. Using residential ZIP codes in medical claims data linked with geographic data sources, we examined how proximity to health care facilities, availability of care providers, and accessible environments were associated with more continuous care. The overall aim of the study was to examine the association between community factors and care continuity in adults aging with CP/SB. Understanding the factors associated with greater COC is important for identifying individuals aging with disability who are at risk of fragmented care, thereby informing appropriate clinical and population-level interventions.

## 2. Materials and Methods

### 2.1. Data Source and Analytical Cohort

Data for this study were obtained from Optum’s de-identified Clinformatics^®^ Data Mart (CDM) Database (2007–2018). This is a nationwide, single private payer, administrative health claims database for over 80 million beneficiaries with commercial and Medicare Advantage health plans in the United States. Data on patient demographic characteristics, inpatient/outpatient records, diagnoses, procedures, and filled prescriptions are available. We used the Clinical Modification (ICD-9-CM) codes from the International Classification of Diseases 9th Edition to identify adults (age 18+ years) with a CP or SB diagnosis (see Supplementary Table S1). Inclusion criteria required that individuals had at least four years of continuous enrolment on the insurance plan to ensure stable membership (*N* = 15,456). Individuals were excluded if they had both a CP and SB diagnosis (*N* = 256) given the lack of clinical plausibility and different disease etiologies. We also excluded 6604 individuals with less than 4 outpatient visits in the year following the date they enrolled on the plan, in order to compute estimates of COC [30]. The final analytic sample consisted of 8596 individuals. To make linkages with data on community characteristics, residential ZIP codes were obtained from CDM. However, when CDM provides ZIP codes to researchers, information on individual-level income, education and race is removed to protect patient privacy. Since secondary data analyses of de-identified datasets cannot be tracked to a human subject, this study was reviewed and categorized as exempt human subjects research by the University of Michigan Institutional Review Board.

### 2.2. Measuring Continuity of Care

Continuity of care was measured using outpatient evaluations and management visits (with unique provider identification numbers) in the one-year period following their enrollment. The Bice-Boxerman COC Index, which captures the degree to which a patient’s outpatient/office visits are concentrated among providers [38], was calculated using the following formula: ((Σ_*i*=1_ *n*_*i*_^2^) − *N/(N(N* − 1)) (where *N* is the total number of visits, and ni is the number of visits with the provider *i*). COC scores range from 0 to 1 with higher scores indicative of a greater share of total visits concentrated within a few unique providers.

The Bice-Boxerman COC score has no inherent meaning and needs to be converted from a continuous to binary or categorical variable for interpretation [30]. Since there is no widely accepted cut-off value, we operationalized COC as a binary variable, consistent with previous studies [39], where individuals with scores above the median (>0.25) were considered to have high COC (high continuity; concentrated care) and those at or below the median were considered to have low COC. To describe the types of providers visited and the frequency of those visits, we also calculated the proportion of all visits in the one-year period to different types of health care providers using the physician-reported specialty in the CDM provider data file (e.g., internal medicine, family/general medicine, obstetrics/gynecology). When clinicians reported more than one specialty, the first reported specialty was used.

### 2.3. Measures of Community Characteristics

Measures of the community environment were obtained from the National Neighborhood Data Archive (NaNDA). NaNDA is a publicly available data archive containing contextual variables derived from a variety of data sources and available at various spatial scales. The measures were linked to the study cohort using residential ZIP codes converted to ZIP Code Tabulation Areas (ZCTA), which are spatial representations of ZIP codes generated by the U.S. Census Bureau with an average population of about 9000 people. NaNDA variables were selected a priori based on associations with COC noted in the literature, or features of the environment that may impact availability and/or accessibility of healthcare services, providers or facilities and affect the ability to maintain a continuous relationship with a set of providers [31]. Data on healthcare establishments included counts of ambulatory care services, hospitals, and residential/skilled nursing facilities in each ZCTA [40]. The availability of broadband internet was based on the number of households with any broadband internet connections per ZCTA [41]. Because public transit may be an important means for accessing health services among people with disability [34], we also included data on the number of public transit stops [42]. For all neighborhood characteristics, we used a measure of per capita density (count divided by total ZCTA population).

Spatial accessibility to healthcare providers, including family medicine doctors (FM), nurse practitioners (NP), medical specialists, and chiropractors, were created using the Variable-distance Enhanced 2 Step Floating Catchment Area (VE2SFCA) method, which includes a distance decay weight accounting for travel time, and a metric of provider to population ratio in each ZCTA [43]. NaNDA measures of neighborhood socioeconomic affluence and disadvantage were used to capture a broader indicator of neighborhood investment and disinvestment [44]. Values range from 0 to 1 with higher scores indicating higher levels of disadvantage or affluence.

In order to account for non-linearity in the relationships between neighborhood resources and health [45,46], all neighborhood variables were operationalized as tertiles (T1 = low, T2 = medium, T3 = high). Due to high collinearity between FM and NP availability, we created a composite measure to capture combinations of these providers as follows: low spatial accessibility (low FM and NP, or low/medium FM/NP), medium spatial accessibility (medium FM and NP, or low/high FM/NP), and high spatial accessibility (high FM and NP, or high/medium FM/NP) (See Supplementary Table S2).

### 2.4. Statistical Analyses

Generalized estimating equations logistic regression models were used to examine the relationship between community factors and odds of high COC, adjusting for individual factors that could increase the risk of worse COC in poor resource neighborhoods. Covariates included age (categorized as age 18–40, 41–64 and 65+ for analysis), sex (male or female), and comorbid conditions (Elixhauser Comorbidity count (range 0–31)) [47]. Year was included to account for both structural improvements in neighborhoods and changes in healthcare policy over the 7-year period in which individuals entered the study cohort. Tests were 2-sided and significance was assessed at *p* < 0.05. All analyses were conducted in SAS version 9.4 (SAS Institute, Cary, NC, USA).

## 3. Results

Descriptive statistics for the study cohort are presented in Table 1 by levels of care continuity. Individuals aging with CP/SB were around 50 years of age on average and 59% were female. Individuals had almost 3 comorbid health conditions on average. The mean COC score was 0.30 overall. Amongst individuals with high COC (categorized as above the median >0.25) the mean Bice-Boxerman score was 0.52, compared to 0.14 for those with low COC (at or below the median). Individuals who had more concentrated care were older than those with low COC (mean age 49.9 years vs. 47.3 years, respectively). Compared to males, females were over-represented in the group with low COC (59% of those with high continuity were female compared to 64% in the low COC group). Co-morbidity burden was similar in those with high or low COC.

Individuals with low and high COC also varied in terms of the characteristics of the communities in which they lived (Table 1). Individuals with high COC were more likely to live in areas with a higher density of residential care/skilled nursing facilities (34.7% vs. 32.0% for high vs. low COC respectively). Compared to those with more continuous care, individuals with low COC were more likely to live in areas with more broadband internet connections (36.6% vs. 30.0%), a greater proximity to a variety of healthcare providers (chiropractors (35.1% vs. 31.5%), medical specialists (35.0% vs. 31.6%), and FM/NP (40.9% vs. 37%, respectively), and in areas characterized as more socioeconomically advantaged (e.g., 37.8% of those with low COC resided in highly affluent communities vs. 28.7% of those with high COC).

**Figure 1.**
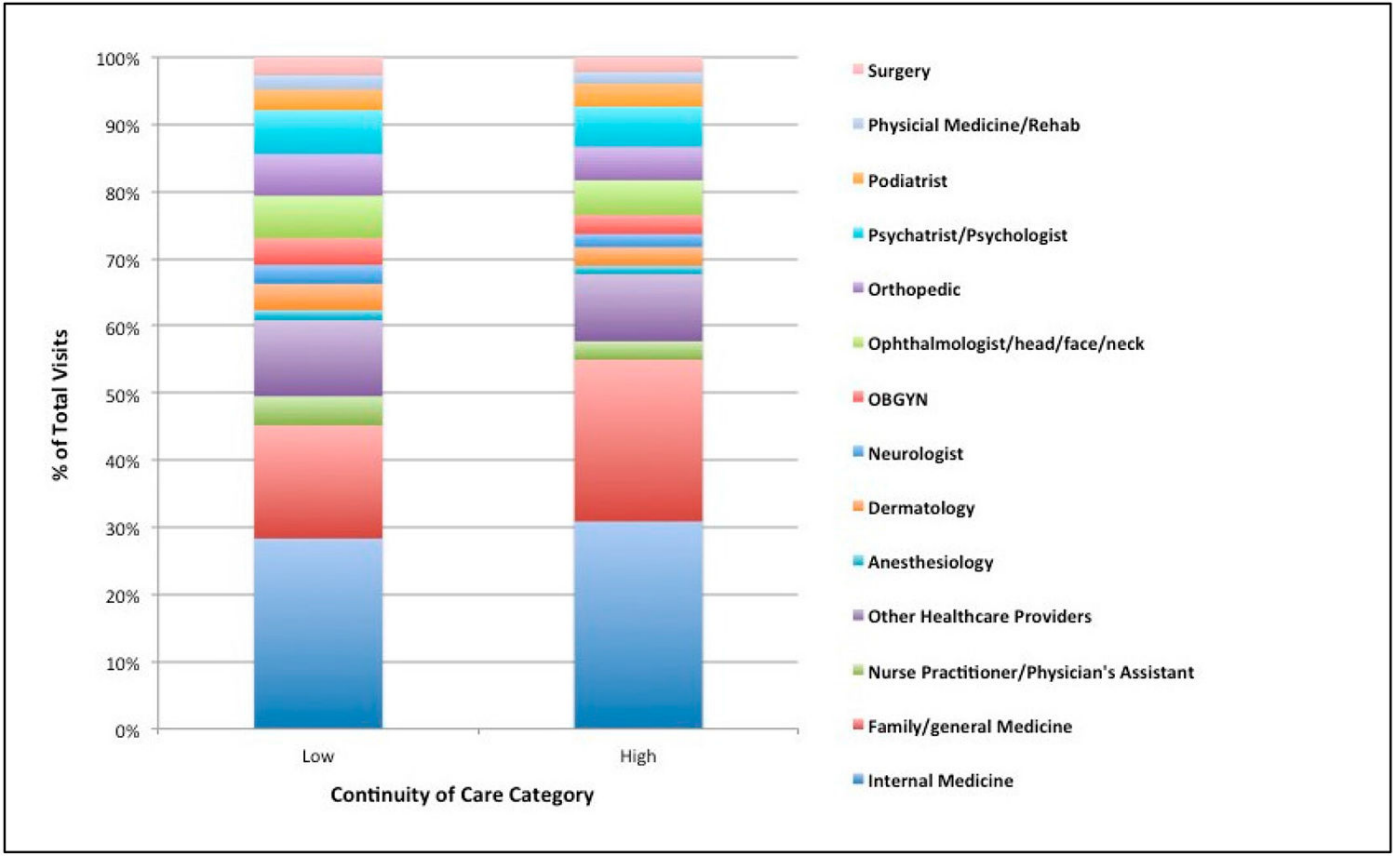
Percentage of visits to different healthcare provider specialties for individuals with cerebral palsy or spina bifida across low and high continuity of care. Note: The order of the colors in the legend corresponds to the order of colors in the bars. Visits to different provider types were examined in the one-year post enrollment date. High COC represents Bice-Boxerman scores above the median (>0.25); low COC = scores at or below the median (≤0.25). OBGYN = Obstetrics and Gynecology.

Figure 1 depicts the distribution of healthcare visits to different specialty types across those with high and low care continuity. Individuals aging with CP/SB, irrespective of COC score, saw more than 14 different provider specialties including orthopedic, neurology, and psychiatry specialties. The most common specialties seen for both groups were family/general medicine (FM/GM) and internal medicine (IM) physicians. However, amongst those with high COC, a greater proportion of total visits were concentrated in these primary care providers. For example, 55% of total visits in those with high COC were to IM and FM/GM specialties compared to 45% for their counterparts with low COC (Figure 1). Compared to those with high COC, a greater share of the health care visits of individuals with low COC were spread between a variety of different types of specialties, including obstetrics/gynecologists (OBGYN) (4% vs. 2.8%), orthopedics (6.2% vs. 5%) and dermatologists (3.9% vs. 2.8%).

Table 2 presents the odds ratios (OR) and 95% confidence intervals (CI) from the multivariable logistic regression model examining the association between community characteristics and the odds of receiving high COC (vs. low COC). The model adjusts for individual age, sex, comorbidity count, and year. After adjusting for individual factors, a greater density of hospitals and residential care facilities was significantly associated with receiving high COC. Residing in areas with a lower density of hospitals was associated with 16% lower odds of high COC (OR for medium vs. high density: 0.84, 95% CI: 0.72–0.98). Compared to areas with a high density of residential care/skilled nursing facilities, areas with low density were associated with 28% lower odds of concentrated care (OR 0.72, 95% CI: 0.59–0.88). Low accessibility of primary care providers (FM and NP) was significantly associated with more concentrated care, net of individual and other community characteristics (low vs. high: OR 1.26, 95% CI: 1.09–1.46). No significant findings were observed for spatial accessibility to medical specialists or chiropractors. Adjusting for health care resources, residence in less affluent areas was associated with higher odds of receiving concentrated care (low vs. high affluence: OR 1.55, 95% CI: 1.29–1.86), with a dose-response relationship observed. No significant associations were found for density of transit stops or broadband internet access and COC (Table 2).

## 4. Discussion

In this large nation-wide cohort of members from large commercial and Medicare Advantage health plans in the United States, we provide one of the first characterizations of COC in adults aging with CP/SB, and highlight how proximity to health care facilities, availability of care providers, and community socioeconomic context are associated with more continuous care.

### 4.1. Continuity of Care in Adults Aging with CP/SB

CP and SB are congenital conditions that result in lifelong limitations in movement. As they age, individuals with CP and SB have progressive motor impairment, excess sedentary behavior, inadequate muscle and bone development, and increased risk for obesity and other high-burden medical conditions (e.g., chronic pain, osteoporosis, and cerebrovascular disease) [48]. These complex care needs create challenges in communicating across different healthcare providers, with increased risk for potentially preventable psychological, cardiometabolic, and musculoskeletal morbidities in adulthood [49]. It is thus not surprising that we found that individuals aging with CP/SB saw a variety of different specialty types and generally had discontinuous care. Extensive work has been done to characterize COC in older adults or those with chronic health conditions, but there is a dearth of research on care patterns in the growing number of adults aging with CP/SB.

In comparison to findings in the general population, our results suggest that individuals aging with congenital disabilities have lower mean COC scores. For example, Medicare/Medicaid patients >65 years with a diagnosis of Diabetes, Congestive Heart Failure, and Chronic Obstructive Pulmonary Disease, had mean Bice-Boxerman COC scores of 0.50, 0.55 and 0.60, respectively [50]. These scores are notably higher than that which we observed in our study, where the mean COC score was 0.30. Amongst those with high continuity, visits were more concentrated in primary care providers (PCP) (i.e., FM, NP and IM). In a previous study of Medicare beneficiaries with multi-morbidity, those reporting a specialist (compared to PCP) as their primary care provider had worse COC score [51,52]. PCP as the central provider may promote better coordination by comprehensively managing conditions and referring to specialists only when necessary [51]. Whereas previous work has largely used Medicare data (>65 years of age), our use of private claims data meant we included a younger cohort of women. For these women, visits to obstetrics/gynecology specialists for reproductive health needs may represent an important source of care and increase the number of specialists seen. Research has highlighted the lack of adequate training to address reproductive health needs of women with disabilities, which might challenge one’s ability to maintain consistent care [53].

### 4.2. Community Resources and Care Continuity

We found that the community context was related to the level of care continuity received. Individuals who lived in areas with more hospitals and residential care facilities received more continuous care than those with limited availability of these resources. Continuous care is more likely when complex medical care can be received in the same location [19]. For those seeing multiple providers, integration of services is more likely when care is confined to a single setting [19]. In hospital settings, there is organization of care, and a care manager, allowing for greater coordination across different types of providers and greater consistency in providers seen. Hospitals also have care coordinators available to manage inpatient and outpatient care, which have been noted in studies to have positive effects on the patient-provider relationship and to reduce coordination problems among patients with complex health care needs [54,55]. Hospital staff often play a role in care plans and arrange for follow-up care, which may not be available in outpatient settings in the community [56]. Similarly, in residential care settings, where the majority of older adults with disabilities reside [57], care coordination can be provided within a single institution.

Adjusting for health care resources, residence in more affluent areas was associated with receiving more fragmented care. Affluent neighborhoods are not just indicative of low disadvantage, but also specific norms such as higher levels of social control and leverage over local institutions that foster environments supportive for health [58]. Healthcare providers often prefer to practice in more affluent areas where residents have more discretionary income, thereby affording individuals with greater choice and shorter travel times to different types of healthcare providers [51,59]. But beyond provider density, COC may be more fragmented in affluent communities where residents tend to have higher levels of health literacy, which may facilitate provider “shopping around” for multiple care providers [60,61]. In many managed care organization health plans, individuals must be proactive and advocate for their healthcare needs from a variety of healthcare providers. Similarly, lower spatial availability of FM/NP was associated with receiving more continuous care. Out of network providers are generally not covered by insurance in managed care organizations making it less likely for individuals who reside in areas with less availability of FM/NP to seek care from multiple different providers outside their communities and insurance networks [22]. Despite expectations, the availability of public transit and broadband internet were not found to be associated with COC after adjusting for other community and individual factors.

### 4.3. Strengths and Limitations

Claims-based measures of care continuity provide a comprehensive record of billed services for beneficiaries aging with congenital disabilities [38]. Claims data provide a more accurate measure of health care encounters across unique physicians and specialty types, mitigating misclassification of the outcome attributed to recall bias in self-reports of care continuity. However, medical diagnoses do not necessarily reflect disability, and we were unable to investigate potential variation in care continuity across the severity of disability in those with CP and SB. Because these data are intended for administrative purposes, there are inherent limitations due to errors in coding. In addition, claims-based measures of COC are not necessarily reflective of health care quality or patient-reported continuity measures [38,62]. The COC index that we used in this study does not capture other important dimensions of coordinated care, including direct communication or co-management between clinicians, and does not consider a patient’s perception of an integrated relationship with their providers. Moreover, calculation of the Bice-Boxerman COC Index requires at least 4 outpatient visits, which meant that we excluded adults with less frequent engagement in the healthcare system who may be in better health. We categorized high and low COC based on the median value of the Bice-Boxerman COC Index, but future research should consider alternate thresholds for high and low COC in this population, perhaps in conjunction with qualitative research that informs the meaning of care continuity in adults aging with CP and SB. The use of private health insurance data in this study limits generalizability and future studies should conduct research using data from publicly insured individuals. By using a measure of spatial accessibility of health care providers that accounted for boundary effects and distance decay functions, we addressed commonplace limitations of density-based measures of spatial accessibility and more accurately reflected the availability of these service providers. However, we were unable to account for the quality of the infrastructure in the built environment. It is plausible that despite a high density of community healthcare facilities, these may be inaccessible for individuals with a physical disability. If this was the case, we likely underestimated the true association between healthcare availability and COC. Our study did not seek to identify causal relationships between community factors and COC, and we were unable to measure the actual use of community resources. Mixed methods research should aim to better understand the relationship between availability, access and use amongst individuals aging with disability. Finally, due to patient confidentiality, information on individual socioeconomic status (SES) was not available when requesting the geographic identifiers in CDM. Although we adjusted for area-level SES, which is a key factor for understanding the socioeconomic experience of populations, neighborhood SES is not a strong proxy for individual SES [63]. Thus, residual confounding by individual SES may result in an over-estimate of the effect estimates in our results.

## 5. Conclusions

Collectively, these findings suggest that individuals aging with CP/SB represent a population particularly prone to fragmented care, perhaps more so than other complex-care populations. Adults living with pediatric onset disabilities in the United States report challenges accessing appropriate care once they transition out of the pediatric care setting [49]. Policy solutions to address care fragmentation have largely focused on shifting payment structures, use of electronic medical records, and decreasing specialty care [64]. Our work contributes to existing calls to promote systematic, coordinated care for people with CP and SB throughout the life course [49] by drawing attention to the spatial distribution of health care resources at the community level, which must also be incorporated when considering ways to address health care disparities in this medically underserved and high-risk population.

## Supplementary Material

Supplementary Materials

## Figures and Tables

**Table 1.** Individual and community characteristics for adults aging with CP/SB by level of continuity of care.

	Overall	High COC	Low COC
N	8596	4221	4375
Continuity of Care, mean (SD)	0.3 (0.19)	0.52 (0.24)	0.14 (0.06)
Individual Characteristics			
Age (years), mean (SD)	48.6 (13.6)	49.9 (14.2)	47.3 (13.9)
Sex, %			
Female	61.6	59.1	64.0
Male	38.4	41.0	36.0
Comorbidity, mean (SD)	2.7 (2.0)	2.6 (2.1)	2.7 (2.0)
Community Characteristics (%)			
Density of Healthcare Resources			
Hospitals			
Low		33.9	32.8
Medium		32.2	34.5
High		34.0	32.7
Residential Care/Skilled Nursing Facilities			
Low		33.0	33.7
Medium		32.3	34.3
High		34.7	32.0
Ambulatory Care Services			
Low		33.8	32.8
Medium		33.4	33.4
High		32.9	33.8
Spatial Accessibility of Healthcare Providers			
Family Medicine/Nurse Practitioners			
Low		42.0	36.6
Medium		21.1	22.4
High		37.0	40.9
Medical Specialists			
Low		34.6	32.1
Medium		33.8	33.0
High		31.6	35.0
Chiropractors			
Low		35.8	30.9
Medium		32.8	34.0
High		31.5	35.1
Socioeconomic Structure			
Affluence			
Low		37.4	29.4
Medium		33.9	32.8
High		28.7	37.8
Disadvantage			
Low		29.6	36.9
Medium		34.8	32.0
High		35.6	31.1
Public Infrastructure			
Density of Public Transit stops			
Low		45.9	45.6
Medium		20.5	21.4
High		33.6	33.0
Density of Broadband Internet Connections			
Low		37.1	29.7
Medium		33.0	33.7
High		30.0	36.6

CP/SB = Cerebral Palsy/Spina Bifida; COC = Continuity of care was calculated in the 1-year post index using the Bice-Boxerman Continuity of Care Index. High COC represents scores above the median (>0.25); low COC = scores at or below the median (≤0.25). Comorbidity was computed based on the Elixhauser Comorbidity Index. SD = standard deviation.

**Table 2.** Logistic Regression Results for the Association between Community Factors and High Continuity of Care for Adults Aging with Cerebral Palsy or Spina Bifida.

Density of Community Characteristics (Ref = High)	Odds Ratio	95% Confidence Interval
Ambulatory Care		
Low	1.10	(0.87, 1.38)
Medium	1.11	(0.95, 1.31)
Hospitals		
Low	0.89	(0.74, 1.07)
Medium	**0.84**	**(0.72, 0.98)**
Residential Care/Skilled Nursing		
Low	**0.72**	**(0.59, 0.88)**
Medium	**0.84**	**(0.73, 0.96)**
Family Medicine/Nurse Practitioners		
Low	**1.26**	**(1.09, 1.46)**
Medium	1.05	(0.92, 1.21)
Medical Specialists		
Low	1.07	(0.91, 1.27)
Medium	1.10	(0.96, 1.25)
Chiropractors		
Low	1.08	(0.95, 1.23)
Medium	1.00	(0.89, 1.12)
Public Transit Stops		
Low	1.09	(0.96, 1.25)
Medium	1.15	(0.99, 1.34)
Broadband Internet		
Low	1.11	(0.94, 1.31)
Medium	0.99	(0.87, 1.13)
Socioeconomic Disadvantage		
Low	0.96	(0.82, 1.13)
Medium	1.15	(0.99, 1.34)
Socioeconomic Affluence		
Low	**1.55**	**(1.29, 1.86)**
Medium	**1.26**	**(1.10, 1.45)**

Modelling odds of high continuity (vs. low continuity). Model is adjusted for age, sex, comorbidity count, and year. Bold values represent statistically significant effects (*p* < 0.05). Ref = Reference group.

## Data Availability

As part of a Data Use Agreement, authors are not allowed to share the data. Upon reasonable request, the first author will provide statistical programming code used to generate results.
