# Supplementary material for: Continuity of Care in Adults Aging with Cerebral Palsy and Spina Bifida: The Importance of Community Healthcare and Socioeconomic Context
Source: Disabilities (Basel). Author manuscript; Available in PMC 2024 Jan 12. (PMC10786460; doi:10.3390/disabilities3020019)
Supplement: Supplementary Materials [file NIHMS1913606-supplement-Supplementary_Materials.pdf]

Supplementary Table S1. International Classification of Diseases, 9th revision, Clinical Modification (ICD-9-CM) codes for cerebral palsy and spina bifida.

Diagnosis Group	ICD-9-CM Code	
**Cerebral Palsy**	3430	Diplegic Infantile Cerebral Palsy
	3431	Hemiplegic Infantile Cerebral Palsy
	3432	Quadriplegic Infantile Cerebral Palsy
	3433	Monoplegic Infantile Cerebral Palsy
	3434	Infantile Hemiplegia
	3438	Other Specified Infantile Cerebral Palsy
	3439	Unspecified Infantile Cerebral Palsy
	33371	Athetoid Cerebral Palsy
**Spina Bifida**	74100	Spina Bifida with Hydrocephalus Unspecified Region
	74101	Spina Bifida with Hydrocephalus Cervical Region
	74102	Spina Bifida with Hydrocephalus Dorsal Region
	74103	Spina Bifida with Hydrocephalus Lumbar Region
	74190	Spina Bifida without Hydrocephalus Unspecified Region
	74191	Spina Bifida without Hydrocephalus Unspecified Region
	74192	Spina Bifida without Hydrocephalus Unspecified Region
	74193	Spina Bifida without Hydrocephalus Unspecified

Supplementary Table S2. Typology used to create the combined indicator variable of spatial accessibility of Nurse Practitioners and Family Medicine physicians.

		Tertile of FM		
Tertile of NP		**Low**	**Medium**	**High**
	**Low**	*Low*	*Low*	*Medium*
	**Medium**	*Low*	*Medium*	*High*
	**High**	*Medium*	*High*	*High*

Abbreviations: FM, Family Medicine; NP, Nurse Practitioner.
Tertiles for each provider type were created from the spatial accessibility of healthcare provider dataset [43].
